# Supplementary material for: Phylogenetic tree shapes resolve disease transmission patterns
Source: Evol Med Public Health. 2014 Jun 9;2014(1):96–108. doi: 10.1093/emph/eou018 (PMC4097963; doi:10.1093/emph/eou018)
Supplement: Supplementary Data [file eou018_supplementary_data.zip › supplement.pdf]

Supplementary Information

Robustness to the nature of the transmission model

We have trained classifiers using simple structural features of phylogenetic trees, and used classification to distinguish between outbreaks with a super-spreader, outbreaks with homogeneous transmission, and outbreaks built from chains of transmission. In the absence of real-world datasets in which the true transmission dynamics are known, we have used simulated outbreaks to train the classifiers. However, this necessitates specifying how the simulations are performed, and determining whether results are robust to our choice of model.

We applied our SVM classifier to simulated phylogenies described in Robinson et al [7], which were derived from a very different model. In that work, dynamic networks of sexual contacts were created based on random graphs with a Poisson distribution (termed 'ER' because they are essentially Erdos-Renyi graphs), and with a distribution of contacts derived from the National Survey on Sexual Attitudes and Lifestyles (NATSAL) [3]. In our simulations, individuals with many contacts reported over the 5-year time frame in the survey had relationships of shorter durations, on average, than individuals who reported only a few contacts. In [7], when epidemics were sampled at one time after the epidemic had spread through the network for some years, the branch lengths, cluster sizes and Colless imbalances of phylogenies did not vary between NATSAL-type contact networks and ER-type networks. This is despite the fact that NATSAL-type networks have individuals with far more contacts than the mean (ie a "core group" of likely super-spreaders). However, when sampling was done over time, the phylogenies from NATSAL-type networks showed higher imbalance than those from ER-type networks, consistent with the work of Leventhal et al [4] on static networks. Here, we used the phylogenies from [7] with a duration of infectiousness $d = 40$ weeks, both under same-time sampling (homochronous) and sampling-through-time (heterochronous). Trees were inferred from simulated sequences using dnaml in the phylip package. Methods are described in detail in [7].

When we classified the homogeneous (ER) and super-spreader (NATSAL-derived) sexual contact networks, we found that the classification was poor on all groups of trees when hosts were sampled at the same time. However, in the heterochronous case, when individuals were sampled throughout the outbreak, the SVM classifiers performed very well with an average specificity and sensitivity of 0.92 (0.045) and 0.92 (0.039) respectively. This is despite the fact that the parameters of the process, contact network, tree inference method and tree sizes were very different from the simulated homogenous, super-spreading, and chain networks the classifiers were trained upon. The basis of the sampling difference is that super-spreaders (or core group members) are likely to be infected early, and so are only included in the sample under heterochronous sampling. This result is consistent with [7], where only trees from heterochronous sampling showed differences in imbalance, branch lengths and cluster sizes between NATSAL and ER networks. The KNN classifier grouped all trees with homogeneous outbreaks, again illustrating that its discriminatory power is much weaker than the SVM approach in distinguishing between these two processes.

Phylogenetic noise

In the results reported in the main text, we extract the true phylogeny from the simulations, based on the fact that we know who infected whom, and can therefore determine the time of the MRCA of all the nodes in the tree. In real outbreaks, this information is of course not available. To determine the extent to which our results are sensitive to phylogenetic noise, we created neighbour-joining trees from the simulations and applied the classification models to these.

We created neighbour-joining phylogenies as follows. The initial (seed) individuals were assigned a random sequence of A, C, T and G of 1000 base pairs in length. When an individual infected another individual, the transmitted sequence was mutated and the number of mutations reflected the time elapsed. As an example, suppose individual i was infected with sequence s_i at time t_{0i} and then infected individuals j and k at times t_{ij} and t_{ik} with $t_{ij} < t_{ik}$. The time elapsed between i's infection and j's infection is $t_{ij} - t_{0i}$. Individual j obtains a sequence s_j which is s_i with m mutations, where m is Poisson-distributed with mean $\mu(t_{ij} - t_{0i})$. Individual k obtains a sequence s_k, which is s_j with a number of mutations distributed as $\text{Poiss}(\mu(t_{ik} - t_{ij}))$. Under this model, k's sequence has

Truth<br>Classification	Hom	SS	Ch
Hom	76 (3)	34 (5)	1 (0.9)
SS	24 (3)	62 (5)	8 (2)
Ch	0 (0.3)	3 (4)	90 (2)

Table S1: KNN classification of neighbour-joining phylogenies. Predictions on the diagonal are correct, and off-diagonal elements show the nature of the mis-classification. Numbers shown are mean over the 10 fold KNN classifiers and numbers in parentheses are standard deviations. As in the main text, most errors are between homogeneous and super-spreader groups, with chain-like outbreaks well resolved.

the mutations occured in host i before i infected j, as well as further variation reflecting the fact that k was infected at a point in time after j. This reflects the simplifying assumption, commonly made, that branching points in the phylogeny are very close to or the same as transmission events [10, 11, 9]. Sequences were sampled at the end of the duration of infection. The mutation rate μ was 6 SNPs per month - biologically unrealistic but required in the current scenario to generate sufficient genetic diversity over the short simulation timescales. If sufficient diversity is present for a phylogeny to be inferred, the mutation rate does not affect the structural properties computed here, and so does not affect the classification. Sampled simulated genome sequences from each outbreak case were aligned and phylogenetic trees for each of the networks were created using matlab's seqpdist and seqneighjoin functions [5]. Trees were constructed using a neighbour-joining method based on the Jukes-Cantor distance between sequences.

We found that phylogenetic noise has an effect on the quality of KNN predictions and a modest effect on the SVM classification. It reduces the specificity and sensitivity of both classifiers, but SVM classification remains good. Table S1 shows a cross-tabulation of the KNN predictions, which overall had 76 (3)% of the homogeneous networks correct, 62 (5)% of the super-spreader networks and 89 (3) % of the chains. Figure S1 shows the ROC when the SVM classifier is applied to homogeneous and super-spreader trees created with the neighbour-joining method. The SVM classifiers had a mean specificity of 97 (3)% and sensitivity of only 15 (14)% but this reflects the automated choice of threshold; results are better illustrated by the ROC.

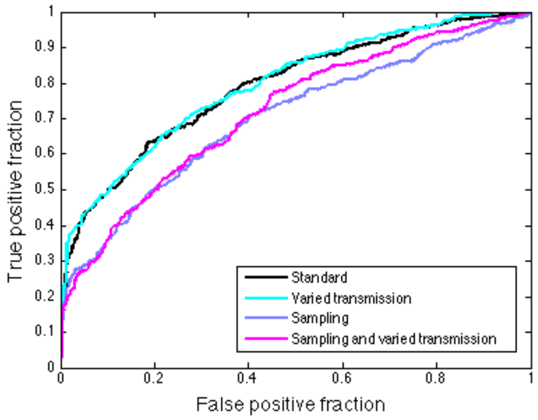

Figure S1: SVM classification (ROC curves) from the neighbour-joining phylogenies. Areas under the curve (AUCS) are mean (std dev): 0.77 (0.15), 0.77 (0.13), 0.71 (0.07) and 0.71 (0.04) for the baseline, variable parameters, variable sampling and variable sampling with variable parameters lines respectively.

Topological summary measures

We considered a number of topological summary measures. These are listed in Table S2 and comprise different combinations of the ladder sizes, imbalance, depths, widths, and numbers of cherry formations [6] in the trees.

Cherry formations, one of the features we examined, were studied in recent work by Frost and Volz [1]. They found expressions for the Sackin imbalance and number of cherries in virus phylodynamic models. A "cherry" is a pair of tips sharing a direct common ancestor, or equivalently, an internal node of the tree with two tip descendants [6]. Frost and Volz found that the number of cherries depended on the ratio of contact rates in two distinct subpopulations, and on the extent to which these subpopulations mixed preferentially within themselves. Also, our model is very different from that of Volz and Frost, in that we have modeled small outbreak trees in non-saturating outbreaks, and their work represents a viral pathogen (HIV) with large enough incidence and prevalence to be modeled with ordinary differential equations. Nonetheless, their approach gives an account of how and why the structures of topologies differ under different underlying epidemiological dynamics, including increased imbalance. Earlier work has also associated imbalance with positive selection and with super-spreaders [2, 4]; intuitively, a lineage corresponding to a super-spreader or one under positive selection would branch more rapidly than others, leaving asymmetric numbers of descendants on the right and left sides of descending sub-trees.

We have used what we term "ladders" in the trees; these are connected sets of internal nodes with a single leaf descendant. Our ladders are closely related to the "caterpillars" studied by Rosenberg [8]. The difference is that a caterpillar terminates in two tips (a cherry), whereas we include ladder formations further up in the tree, terminating in more complex sub-trees. See Figure **??** for an illustration. In our outbreaks, super-spreaders were infected early, and their sequences continued to mutate throughout the outbreak. As such, the signatures of super-spreading were likely to occur in the "ancestral" sequences – those of the super-spreader and their secondary infections prior to the mutations that occurred during the remainder of the outbreak. This motivates the use of structural features that occur internally in the tree, in contrast to cherries which reflect the dynamics among the most recently observed sequences. Cherry numbers would also be expected to change depending on sampling, though an exploration of how sampling affects phylogenetic structure is beyond the scope of this work.

Model parameters

Figure S2 shows the distribution of infectious period in the model; this was the same for both types of networks. The association between topological structures and transmission via super-spreaders is dependent on these parameters, and in particular, when the duration of infection becomes quite long (greater than 2 years) and the value of R decreases (below 1.3), the associations we report become weaker as a result of the very spread-out nature of transmission with these parameters. However, the results were robust to variation of the parameters such that β/D was uniform in [1.25, 2.5].

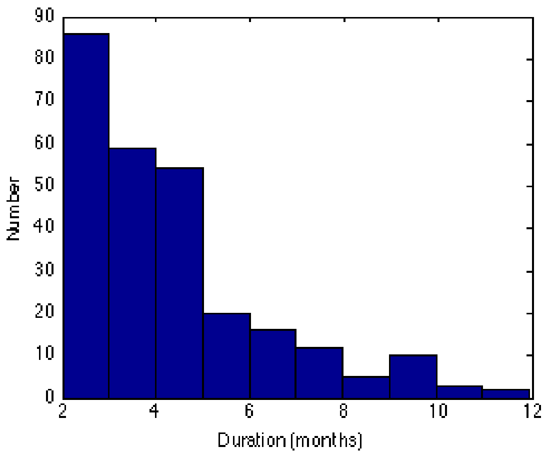

Figure S2: Distribution of lengths of infectious period in the transmission model

Name	Description	Relation to outbreak type
Imbalance	Colless's I, defined in main text	Higher for super-spreader outbreaks
IL portion	Portion of internal nodes with one leaf descendant	Higher for super-spreader outbreaks
$\max(l/N)$	Maximum ladder length / number of leaves	Higher for super-spreader outbreaks
Scaled depth	Maximum depth / number of leaves	Higher; more variable for super-spreader outbreaks and anti-correlated with width/depth
Δw	As in main text; max difference in widths at successive depths	Lower for super-spreader outbreaks
Maximum width/N	Maximum width over number of leaves	Lower for super-spreader outbreaks
Cherry number/N	Number of cherries over number of leaves	Slightly lower and more variable for super-spreader outbreaks
Staircase-ness 1	Portion of imbalanced nodes	much lower for chain outbreaks
Staircase-ness 2	Mean ratio of min/max number descendants	much lower for chain outbreaks
Sackin imbalance	Mean path length from tip to root	Slightly higher in super-spreader outbreaks and lower in chains

Table S2: Summary measures for phylogenetic trees. We have included a comment about the relationship of the summary to the outbreak type, but this does not capture the relationships between different measures (which are captured by the computational classification approaches).

Tree size

While the structural features were normalised linearly, we did not ensure that they were scaled to be independent of tree size because the two real outbreaks were close in size. It is challenging to characterise the asymptotic expected value of structural features (see for example [8]). For outbreaks of the size reported here, asymptotic results may not be very meaningful in any case. Furthermore, most of the work that has been done has focused on trees derived from one of several common simple models, including the Yule model. These may not be good descriptions of trees from densely sampled outbreaks, because in densely sampled outbreaks, the fact that infection processes are not memory-less affects the shape of the trees. When there is a new infection, the infectious period of the original host does not "reset", as would match the assumptions of homogeneous branching process models. Rather, the original host is likely to recover sooner than the newly-infected host, unless the infectious period is truly memory-less. For these reasons, we are uncertain how the structural features should scale on average with tree size, either in the Yule model or in a model adjusting for this asymmetry in the infectious period. We computed the structural features and show the dependence on tree size in Figure S3. We found that while most features do not change dramatically with size, some do. This is likely one reason that classification performed poorly when only a few isolates had been observed. If we were interested in analysing much larger trees than those here, then it would be necessary to use summary features that capture more higher-resolution information about the tree at different scales than the very simple summary features we have used in any case. Simulation results such as the ones reported here could be the basis of conjectures as to how the structural summaries scale with tree size, but this analysis is beyond the scope of this work.

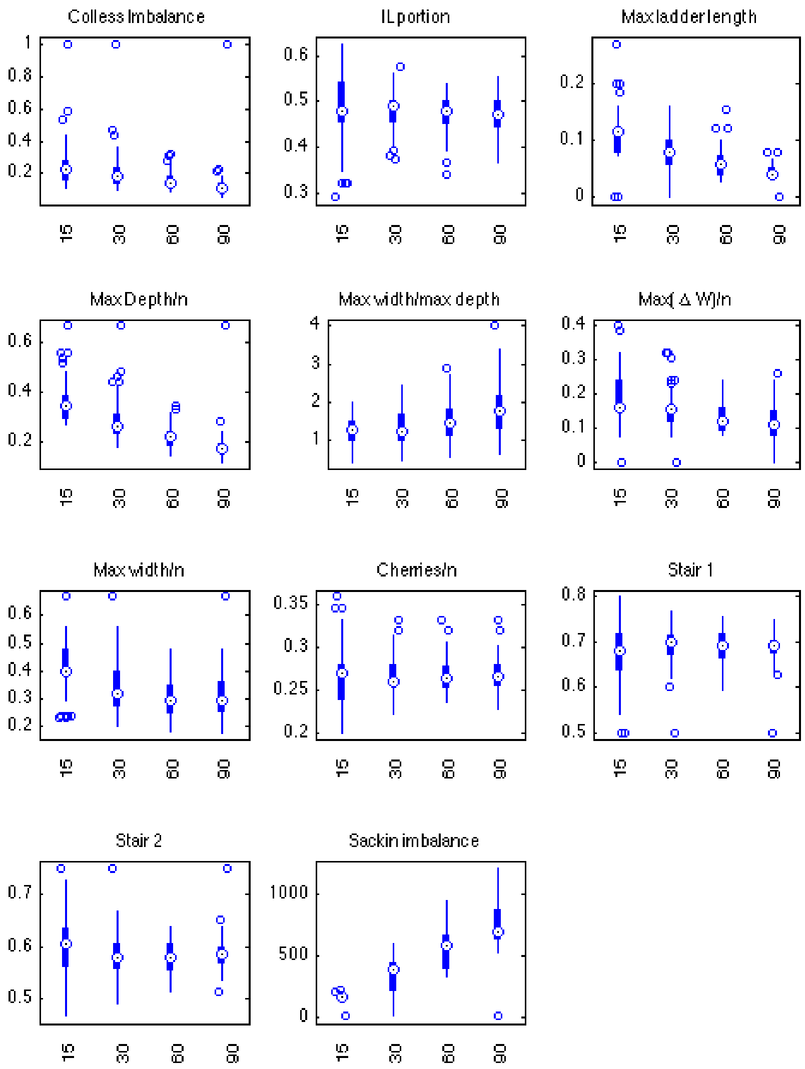

Figure S3: The structural features of trees, showing the dependence of the results on the number of tips included. Trees were derived from the homogeneous networks with the baseline parameter values reported in the main text.

References

[1] Simon DW Frost and Erik M Volz. Modelling tree shape and structure in viral phylodynamics. *Philosophical Transactions of the Royal Society B: Biological Sciences*, 368(1614), 2013.

[2] Stephen B. Heard. Patterns in Phylogenetic Tree Balance with Variable and Evolving Speciation Rates. *Evolution*, 50(6):2141–2148, 1996.

[3] A. M. Johnson, C. H. Mercer, B. Erens, S. Copas, A. J. an d McManus, K. Wellings, K. A. Fenton, C. Korovessis, W. Macdowal l, K. Nanchahal, S. Purdon, and J. Field. Sexual behaviour in britain: partnerships, practices, and hiv risk behaviours. *Lancet*, 358(9296):1835–1842, December 2001.

[4] G.E. Leventhal, R. Kouyos, T. Stadler, V. von Wyl, S. Yerly, J. Böni, C. Cellerai, T. Klimkait, H.F. Günthard, and S. Bonhoeffer. Inferring epidemic contact structure from phylogenetic trees. *PLoS Computational Biology*, 8(3):e1002413, 2012.

[5] MATLAB. *version 7.14.0.739 (R2012a).* The MathWorks Inc., Natick, Massachusetts, 2012.

[6] Andy McKenzie and Mike Steel. Distributions of cherries for two models of trees. *Mathematical biosciences*, 164(1):81–92, 2000.

[7] Katy Robinson, Nick Fyson, Ted Cohen, Christophe Fraser, and Caroline Colijn. How the dynamics and structure of sexual contact networks shape pathogen phylogenies. *PLoS computational biology*, 9(6):e1003105, 2013.

[8] Noah A Rosenberg. The mean and variance of the numbers of r-pronged nodes and r-caterpillars in yule-generated genealogical trees. *Annals of Combinatorics*, 10(1):129–146, 2006.

[9] T. Stadler, R. Kouyos, V. Von Wyl, S. Yerly, J. Böni, P. Bürgisser, T. Klimkait, B. Joos, P. Rieder, D. Xie, et al. Estimating the basic reproductive number from viral sequence data. *Molecular Biology and Evolution*, 29(1):347–357, 2012.

[10] Tanja Stadler and Sebastian Bonhoeffer. Uncovering epidemiological dynamics in heterogeneous host populations using phylogenetic methods. *Philosophical Transactions of the Royal Society B: Biological Sciences*, 368(1614), 2013.

[11] E.M. Volz, J.S. Koopman, M.J. Ward, A.L. Brown, and S.D.W. Frost. Simple epidemiological dynamics explain phylogenetic clustering of hiv from patients with recent infection. *PLoS Computational Biology*, 8(6):e1002552, 2012.
